# Concurrent Courses: Exploring the Synchronous Occurrence of Hepatic Myelolipoma and Hepatocellular Carcinoma

**DOI:** 10.7759/cureus.84216

**Published:** 2025-05-16

**Authors:** Syed Yasir Afaque, Lucy Gossage, Cheika Kennedy, Samiya Ibrahim, Katie Walter

**Affiliations:** 1 Oncology, Cambridge University Hospitals NHS Foundation Trust, Cambridge, GBR; 2 Oncology, Nottingham University Hospitals NHS Trust, Nottingham, GBR; 3 Radiology, Nottingham University Hospitals NHS Trust, Nottingham, GBR; 4 Histopathology, Nottingham University Hospitals NHS Trust, Nottingham, GBR; 5 Colorectal Surgery, Nottingham University Hospitals NHS Trust, Nottingham, GBR

**Keywords:** collision tumours, hepatopancreatic surgery, histopathology, oncology, radiology

## Abstract

Myelolipomas are benign, nonfunctioning neoplasms that most commonly arise in the adrenal glands and constitute a recognisable subset of adrenal masses. They are primarily composed of haematopoietic cells and fatty tissues. Hepatic myelolipoma is extremely rare, with only 11 cases previously recorded in English medical literature. It is usually an incidental finding. Hepatic myelolipomas are most often identified by ultrasound (US), computed tomography (CT) and magnetic resonance imaging (MRI), and diagnosis is confirmed with histopathology. In this case report, we describe an exceptionally uncommon case in which a patient presented with aberrant and nonspecific symptoms. He was discovered to have a massive hepatic myelolipoma, as well as elements of hepatocellular carcinoma (HCC). He underwent tumour resection after being carefully reviewed by the sarcoma multidisciplinary team (MDT).

## Introduction

Myelolipoma is an uncommon non-malignant tumour, usually arising within the adrenal gland, composed of adipose tissue and bone marrow-derived haematopoietic elements. Adrenal myelolipomas are often diagnosed incidentally and, hence, are often known as adrenal incidentalomas [1]. Patients diagnosed with hepatic myelolipomas have an average age of 48.8 years, ranging from 20 to 76 years, indicating that the condition does not target a specific age group. Currently, it is unclear from the literature which environmental or genetic factors predispose people to developing myelolipomas. Adrenal myelolipomas may be more common in patients with endocrine disorders, such as Cushing's syndrome or Conn's syndrome. This implies that the aetiology of myelolipomas may involve hormonal imbalances or disruptions in the endocrine system.

In this report, we describe the simultaneous occurrence of a hepatic myelolipoma and hepatocellular carcinoma (HCC) in one patient, identified incidentally. This is only the third occurrence documented in medical literature [2,3]. We discuss the clinical, radiological and pathological aspects of this case and provide a brief overview of the field of hepatic myelolipoma. This report was previously presented as a poster at the 2024 British Sarcoma Group Conference on February 27, 2024.

## Case presentation

**Figure 1:**
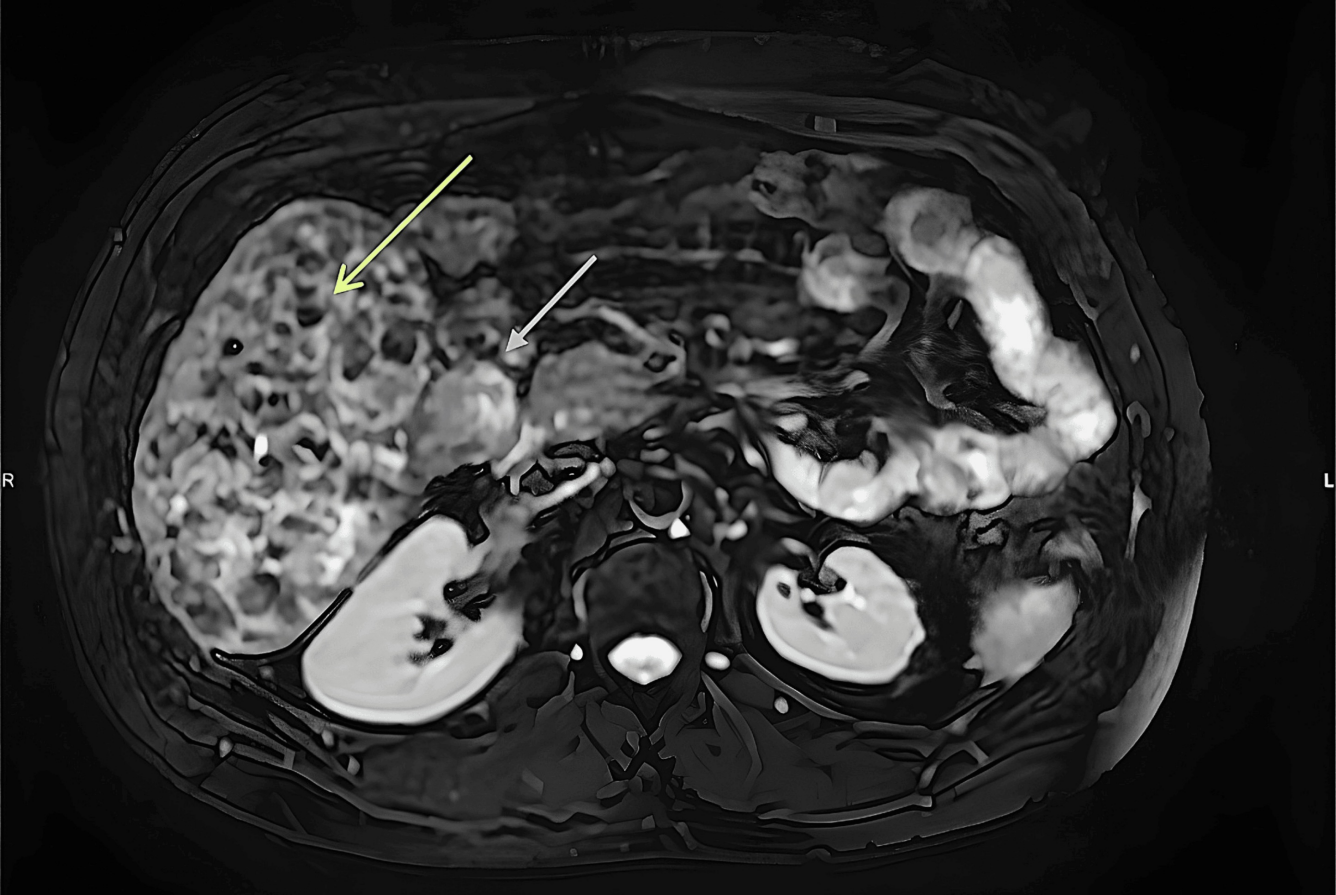
Non-contrast whole-body MRI (axial T2-weighted through the upper abdomen) Showing a 15 cm heterogeneous exophytic liver lesion (shown in yellow arrow) with peripheral nodules, the largest being 4 cm, arising from the liver, in contact with the duodenum and right kidney and close to the inferior vena cava (shown in white arrow) MRI: magnetic resonance imaging

A healthy White British man in his early 30s who occasionally drank alcohol within limits (under 14 units per week) presented to his general practitioner (GP) with pain under the left scapular region that was affecting his ability to lift at work. The pain was localised and affected his daily activities for 2-3 months. He had no notable prior medical conditions and no identifiable risk factors for liver disease. On clinical examination, there was mild tenderness at the right upper abdominal quadrant with no associated organomegaly or palpable masses. Initial assessments and a chest X-ray requested by the GP were insignificant. However, the patient was troubled by persistent symptoms, so he arranged a private, whole-body magnetic resonance imaging (MRI) (Figure 1).

Investigations

**Figure 2:**
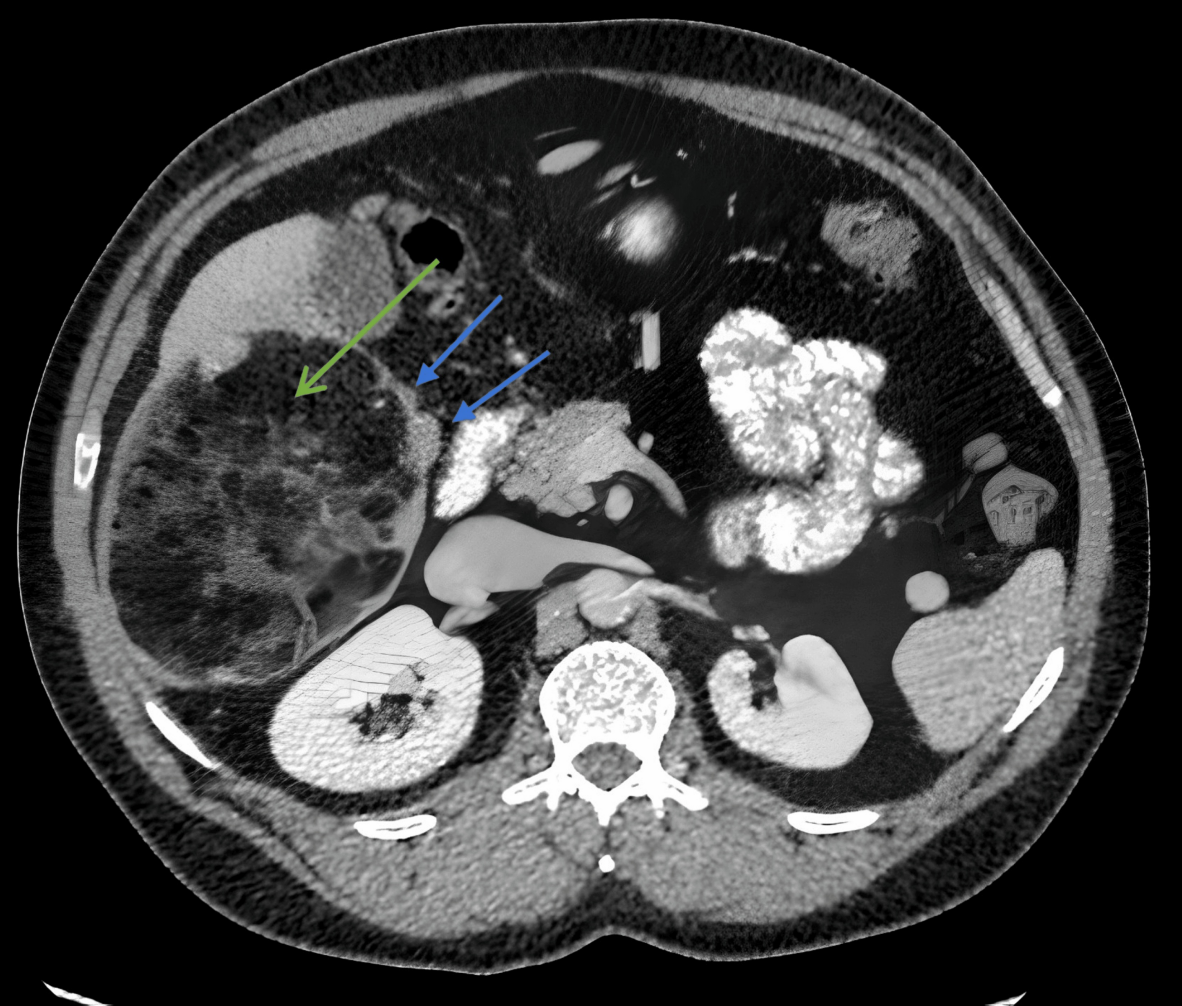
Post-contrast portal venous-phase CT of the abdomen A solitary, large, predominantly fat-density multiseptated lesion arising from and expanding the caudal aspect of the right lobe of the liver (shown in green arrow) with soft tissue mural nodules (shown in blue arrow) CT: computed tomography

The whole-body non-contrast MRI (Figure 1) revealed an incidental 15 cm fat-containing abdominal mass with peripheral nodules, the largest being 4 cm, arising from the liver, in contact with the duodenum and right kidney and close to the inferior vena cava. No other abnormal findings were noted. At this point, the patient was referred to the retroperitoneal sarcoma multidisciplinary team (MDT) who advised a computed tomography (CT) of the chest, abdomen and pelvis and biopsy to identify the origin and nature of the mass (Figure 2).

Initial blood tests were unremarkable other than slightly elevated alanine transaminase and aspartate transaminase, which were 72 U/L and 40 U/L, respectively. Haemoglobin and bilirubin levels were within the normal range. Contrast-enhanced CT images showed a solitary, large, predominantly fat-density multiseptated lesion arising from and expanding the caudal aspect of the right lobe of the liver, which measured 11.3 × 9.8 cm axially and extended for 14.6 cm in craniocaudal dimension. The lesion contained soft tissue mural nodules, the largest being 39 mm. Fatty hepatic lesions in segment 5/6 were stable in size with exophytic and endophytic nodules, which demonstrated arterial-phase hyperenhancement but no washout. Low attenuation in segment 4 on the arterial-phase acquisition corresponded to a perfusion anomaly when correlated with previous MRI.

**Figure 3:**
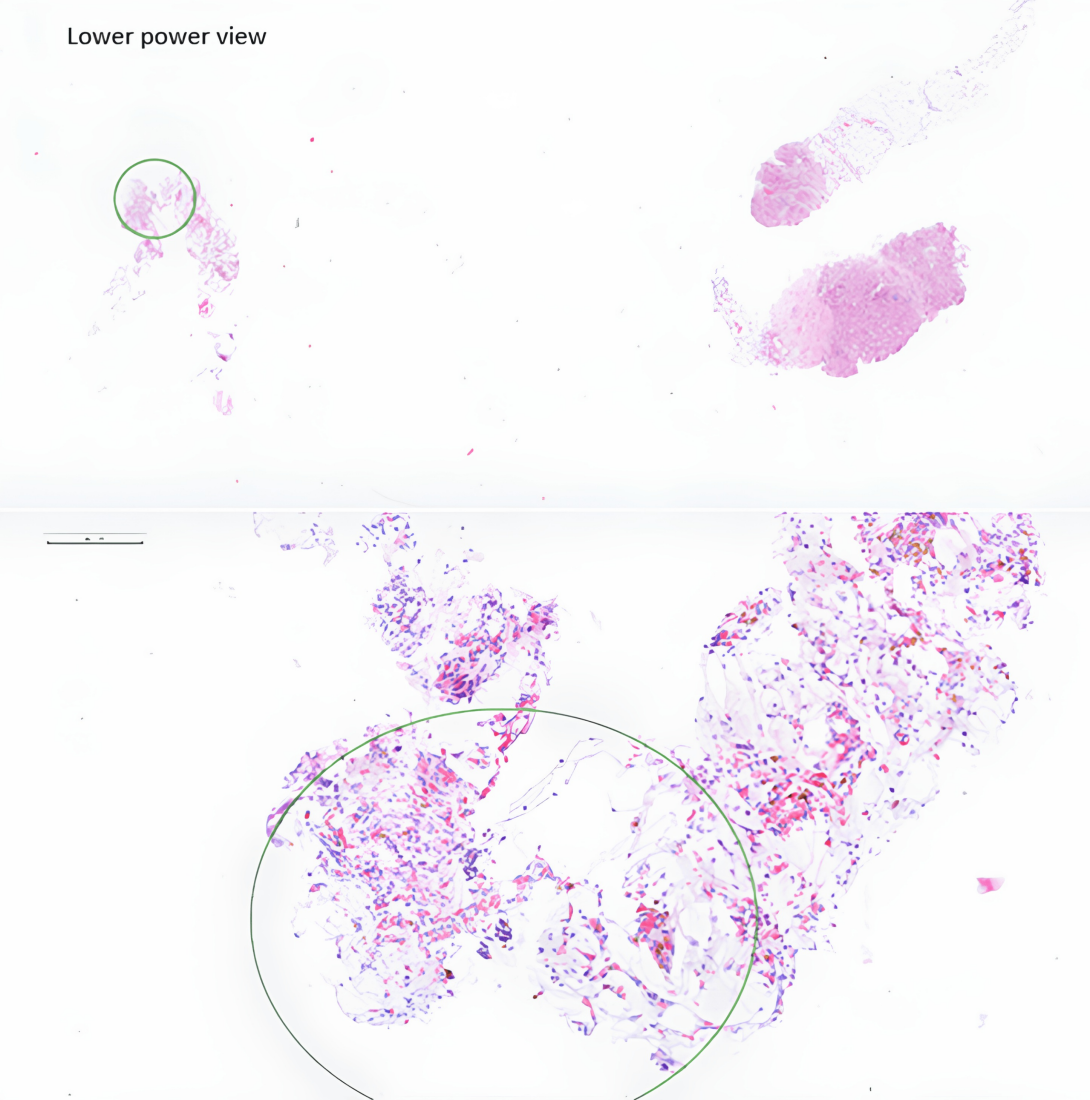
Liver biopsy Small cores of hepatic tissue with focal cholestasis, with attached mature adipose tissue showing fat necrosis and erythropoietic groups without obvious megakaryocytes, myelolipoma (circled in green)

Biopsy revealed a few small cores of hepatic tissue with focal cholestasis, with attached mature adipose tissue showing fat necrosis and erythropoietic groups without obvious megakaryocytes. There was no evidence of atypia, malignancy, pleomorphism and mitotic figures (Figure 3). The adipocytes stained negative for HMB45 and Melan A. The fluorescence in situ hybridisation (FISH) test for MDM2 was negative. Within the areas of bone marrow differentiation, immunohistochemistry for myeloperoxidase was positive, glycophorin A highlighted erythroid cells, cluster of differentiation 42B (CD42B) highlighted megakaryocytes and cluster of differentiation 34 (CD34) highlighted occasional myeloblasts and lymphoblasts. These features were in keeping with a myelolipoma arising in the liver (Figure 3).

Considering the imaging and histopathology, it was felt that a myelolipoma would be a unifying diagnosis. However, there were concerns that the nodules at the periphery of the lesion were atypical and may represent a second pathology.

**Figure 4:**
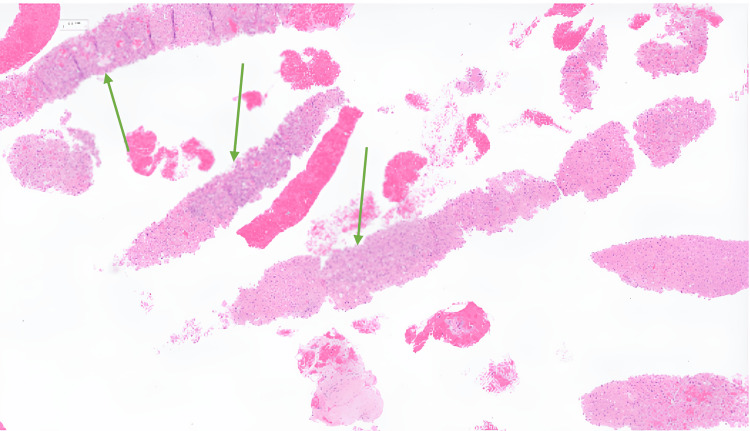
Biopsy of exophytic component Linear fragments of hepatocellular carcinoma (shown in green arrow)

A second biopsy of the exophytic component was arranged. This biopsy consisted of multiple fragments and cores of tissue consisting of bland liver cells but with an abnormal architecture, including prominent cholestatic rosettes and adenoid or pseudo-acinar arrangement. It consisted of abundant cholestasis, and the cytoplasm showed vacuolation, which was probably hydropic change, although there may also be some small-droplet fat. No portal tracts were present. Immunohistochemistry showed positive staining for hepatocyte antigen and glypican 3, and CD34 showed diffuse sinusoidal staining. Reticulin showed an abnormal pattern with pocketing, while beta-catenin showed nuclear staining, and glutamine synthetase showed diffuse overexpression. The features were of a primary hepatocellular tumour, and appearances were most consistent with a well-differentiated HCC. No myelolipoma was seen in this biopsy (Figure 4).

**Figure 5:**
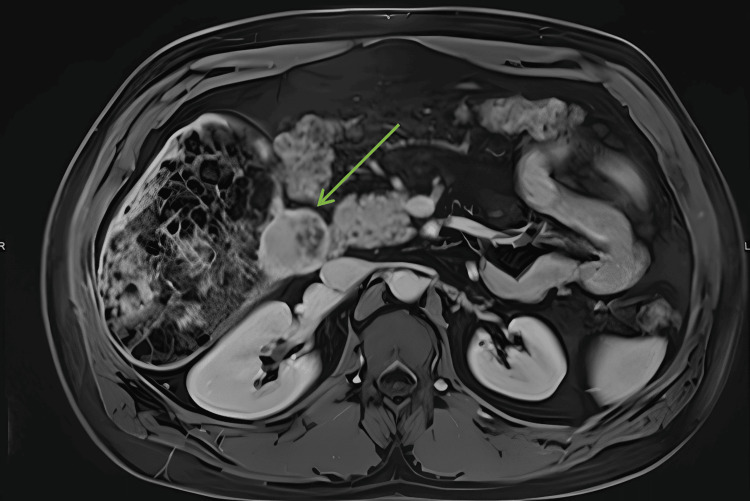
Repeat contrast-enhanced MRI of the liver Growth of mural nodules, especially the medial lesion adjacent to the head of the pancreas (shown in green arrow) MRI: magnetic resonance imaging

A repeat contrast-enhanced MRI of the liver, five months after the initial whole-body MRI, showed both the segment 5/6 fat-containing lesions with progressive mural nodularity (Figure 5). These exophytic and internally projecting nodules demonstrated arterial-phase hyperenhancement; however, there was no convincing washout. These areas also demonstrated true diffusion restriction. The gallbladder, pancreas, spleen, right kidney and adrenals were normal. The left kidney showed mild atrophy. There was no regional lymphadenopathy and no free fluid.

Differential diagnosis

A variety of differential diagnoses were examined prior to the definitive diagnosis. Due to the substantial fatty component of the tumour on radiology, these included hepatic liposarcoma and angiomyolipoma. Biopsy and immunohistochemistry, however, revealed a benign hepatic myelolipoma. Additionally, the presence of haematopoietic elements is an important distinguishing feature in this differential diagnosis. Furthermore, the absence of cellular atypia, pleomorphism and mitotic figures helps to rule out malignant processes.

A second multidisciplinary team opinion was sought regarding the suspicious exophytic nodules observed at the tumour's perimeter, which validated an additional diagnosis of well-differentiated hepatocellular carcinoma, classified as Barcelona clinic liver cancer (BCLC) stage A and Child-Pugh class A, with no radiological or histological evidence of metastatic disease.

Treatment

The patient was referred to the hepatic, pancreatic and biliary multidisciplinary team who advised hemihepatectomy with cholecystectomy, which the patient agreed to after thorough counselling. Surgery was uneventful, and the tumour was resected with R0 margins. The distance between the two protruding nodules was 10 mm. These two extrahepatic tumour nodules were surrounded by a capsule and the subcapsular position of the liver. The third tumour mass arose within tumour 2 beneath the capsule. The adrenals were spared, and the rest of the liver was normal with no gross abnormality.

**Figure 6:**
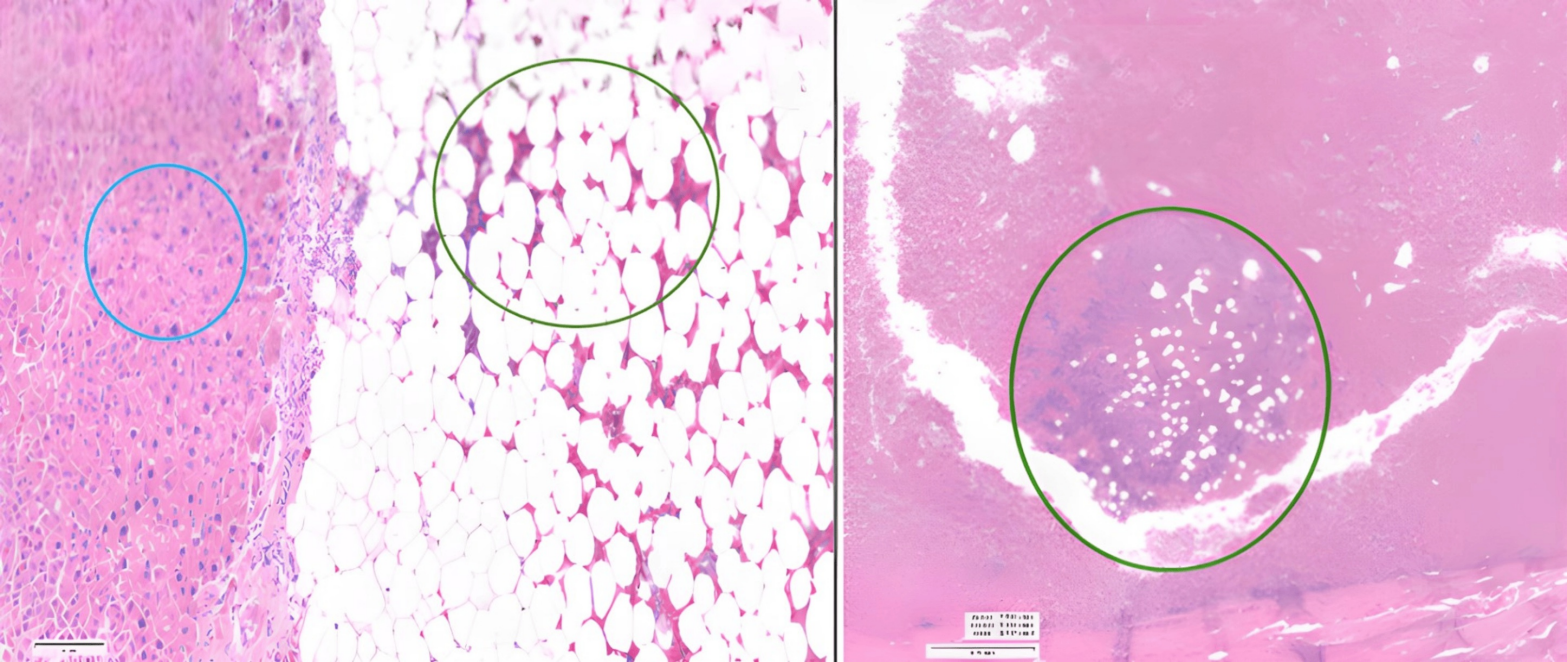
Postoperative H&E sections Well-differentiated hepatocellular carcinoma with solid, trabecular and pseudo-acinar patterns (circled in blue) and myelolipoma containing mature adipose tissue with scattered areas composed of mature bone marrow with foci of osseous metaplasia (circled in green)

Postoperative histology showed the synchronous occurrence of HCC and myelolipoma with mixed HCC (T2) infiltrating and surrounding the adjacent myelolipoma (pT2 N0 Mx G1 R0) (Figure 6). Tumour 1 cut section showed haemorrhage and green pigment. Tumour 2 indicated myelolipoma. The background liver showed no cirrhosis. The margin of excision of tumour 2 was not involved. The tumour was encapsulated and was in the subcapsular position of the liver. It had a variable appearance, and one component was rich in fat. The other was more solid and haemorrhagic.

Sections of the gallbladder showed mild chronic inflammation, focal Rokitansky-Aschoff sinus formation and cholesterolosis. There was no evidence of dysplasia or malignancy.

Immunohistochemistry showed positive glutamine synthetase (strong and diffuse), hepatocyte, CD34 and glypican 3 (patchy) and negative alpha-fetoprotein (AFP). Ki67 showed a low proliferation ratio of less than 1%. Reticulin showed an abnormal architectural pattern in the tumour nodules.

The larger mass (tumour 2) was composed predominantly of mature adipose tissue with scattered areas composed of mature bone marrow with occasional foci of osseous metaplasia. No adrenal gland elements were identified. Within the areas of bone marrow differentiation, immunohistochemistry for myeloperoxidase was positive, glycophorin A highlighted erythroid cells, CD42B highlighted megakaryocytes and CD34 highlighted occasional myeloblasts and lymphoblasts. These features were in keeping with a myelolipoma arising in the liver as previously described.

Follow-up and outcome

The patient had an uncomplicated postoperative recovery and is currently under surveillance. As investigations excluded evidence of metastatic disease or malignancy, adjuvant chemotherapy was not indicated. This will involve monthly MRI scans for the first two years, followed by an MRI every six months for the subsequent three years as part of secondary surveillance. Thereafter, follow-up will continue with ultrasound (US) scans at six-month intervals.

## Discussion

Myelolipoma is a rare benign tumour of mesenchymal origin composed of fat and haematopoietic cells. As such, it resembles a site of extramedullary haematopoiesis. The adrenal gland is the most common site of myelolipomas. Extra-adrenal myelolipomas are very rare. They have previously been reported in the parietal pleura, presacral area and retroperitoneum [4,5].

In the present study, we report a case of the synchronous occurrence of adjacent hepatic myelolipoma and hepatocellular carcinoma in one patient, identified incidentally when the patient paid for a private whole-body MRI for unrelated symptoms.

Lipomatous masses of hepatic origin, such as lipomas, angiomyolipomas, metastatic dermoid tumours, Langerhans cell histiocytosis and focal fat infiltration, should be separated from hepatic myelolipoma [6]. Hepatocellular carcinomas and hepatic adenomas can also occasionally exhibit areas of fatty degeneration [7]. Myelolipomas are benign tumours; however, they can develop symptoms and grow. Therefore, if the tumour is symptomatic or difficult to diagnose, surgical intervention becomes a necessity. The third greatest mortality rate among cancer patients for whom surgical resection is the most frequent and efficient therapeutic approach is seen in individuals with hepatocellular carcinoma, the sixth most prevalent malignancy in the world [8,9]. To totally remove the hepatic myelolipoma and hepatocellular carcinomas, the patient underwent a right hemihepatectomy. A cholecystectomy was also done because the gallbladder was adjacent to the tumour. The surgical specimen's histopathological and immunohistochemical investigations supported the diagnosis of synchronous hepatic myelolipoma and hepatocellular carcinoma.

This case illustrates collision tumours, which are extremely rare and are made up of two or more different neoplasms that coexist with little or no link. They are frequently discovered incidentally during pathological studies of resected specimens, posing significant diagnostic challenges [10]. Atypical features in rare tumours pose a diagnostic challenge for both radiology and pathology. In this instance, the sampling bias of the fatty component gave the multidisciplinary team reassurance that the lesion was benign (BCLC stage A). The rapid interval changes of the mural nodules changed the threshold of suspicion and ensured appropriate management. Hepatocellular carcinoma and myelolipoma rarely occur simultaneously. To our knowledge, this is only the third described case of synchronous hepatic myelolipomas and hepatocellular carcinoma.

While the histopathology of the myelolipoma in this patient matched the radiology of the fatty lesion, the multidisciplinary team was concerned that the medial nodule was atypical; therefore, a second biopsy of this was performed.

## Conclusions

This case highlights the importance of recognising collision tumours, such as hepatic myelolipoma coexisting with hepatocellular carcinoma, when evaluating complex hepatic lesions with both fatty and solid components. The awareness of such rare entities is essential to avoid misdiagnosis, particularly given the risk of sampling bias in heterogeneous lesions. Radiologically guided biopsies must be meticulously targeted to the most suspicious areas, using advanced imaging modalities such as multiphasic MRI with fat suppression and contrast enhancement to accurately characterise lesion components.

A joint multidisciplinary team (MDT) review is imperative to correlate histological and radiological findings, ensuring that biopsy samples are representative and sampling errors are minimised. The MDT, comprising hepatologists, hepatic imaging specialists, interventional radiologists and hepatobiliary pathologists, should confirm that histology aligns with imaging features. We recommend developing a diagnostic algorithm that incorporates key imaging red flags, targeted biopsy strategies and defined MDT review pathways to enhance diagnostic accuracy and prevent the under-recognition of rare but clinically significant collision tumours.

## References

[1] Calissendorff J, Juhlin CC, Sundin A, Bancos I, Falhammar H (2021). Adrenal myelolipomas. Lancet Diabetes Endocrinol.

[2] Xu SY, Xie HY, Zhou L, Zheng SS, Wang WL (2016). Synchronous occurrence of a hepatic myelolipoma and two hepatocellular carcinomas. World J Gastroenterol.

[3] Costa AF, Thipphavong S, Arnason T, Stueck AE, Clarke SE (2018). Fat-containing liver lesions on imaging: detection and differential diagnosis. AJR Am J Roentgenol.

[4] Decmann Á, Perge P, Tóth M, Igaz P (2018). Adrenal myelolipoma: a comprehensive review. Endocrine.

[5] Nishizaki T, Kanematsu T, Matsumata T, Yasunaga C, Kakizoe S, Sugimachi K (1989). Myelolipoma of the liver. A case report. Cancer.

[6] Xin H, Li H, Yu H, Zhang J, Peng W, Peng D (2019). MR imaging to detect myelolipomas of the liver: a case report and literature review. Medicine (Baltimore).

[7] Van Hoe L, Gryspeerdt S, Van Eycken P, Baert AL, Marchal G (1994). Myelolipoma in a hepatocellular carcinoma: CT-pathologic correlation. AJR Am J Roentgenol.

[8] Condon WB, Safarik LR, Elzi EP (1965). Extramedullary hematopoiesis simulating intrathoracic tumor. Arch Surg.

[9] Yoshikawa J, Matsui O, Takashima T (1988). Fatty metamorphosis in hepatocellular carcinoma: radiologic features in 10 cases. AJR Am J Roentgenol.

[10] Ferlay J, Soerjomataram I, Dikshit R (2015). Cancer incidence and mortality worldwide: sources, methods and major patterns in GLOBOCAN 2012. Int J Cancer.

